# Chronic kidney disease screening to reduce cardiovascular risk: a call to action

**DOI:** 10.1016/j.ajpc.2025.101380

**Published:** 2025-12-11

**Authors:** Erin D. Michos, David Cherney, Pam Kushner

**Affiliations:** a Division of Cardiology, Department of Medicine, Johns Hopkins University School of Medicine, Blalock 524-B, 600 N Wolfe Street, Baltimore, MD 21287, USA; b Division of Nephrology, University Health Network, Department of Medicine, University of Toronto, 585 University Ave, 8N-845, Toronto, Ontario M5G 2N2, Canada; c Department of Family Medicine, University of California at Irvine, 1001 Health Sciences Road, Irvine, CA 92697, USA

**Keywords:** Chronic kidney disease, Cardiovascular disease, Heart failure, Screening, Preventive cardiology, Risk assessment, Risk factors

## Abstract

Chronic kidney disease (CKD) has a high global prevalence, affecting around 1 in 7 adults in the United States; however, most adults with CKD are unaware that they have the condition. Diagnosis and treatment of CKD is essential due to the associated increased morbidity and mortality, including increased risk of cardiovascular disease (CVD) and heart failure. Importantly, people with CKD are more likely to die from CVD than progress to end-stage kidney disease. Dual evaluation of estimated glomerular filtration rate (eGFR) and urinary albumin-to-creatinine ratio (UACR) is essential to determine the level of risk and to guide appropriate treatment. Although abnormalities in both eGFR and UACR can be modifiable risk factors for CKD progression and adverse CV outcomes, there is evidence of underuse of this dual screening for CKD. However, for patients with diagnosed CKD, striking reductions in cardiorenal risk may be achieved by combining appropriate evidence-based therapies. Current approaches to management of CKD involve the use of multiple therapies that target different pathological pathways to reduce cardiorenal risk. Therefore, we raise a call to action to improve the standard of care for early diagnosis and management of CKD, to minimize the risk of disease progression and complications, reduce CV risk, and ultimately improve patient outcomes. Alongside primary care clinicians, cardiologists can also lead the way for preventive efforts and implementation of guideline-directed therapies that can reduce the risk of both CKD progression and adverse CV outcomes.

**Figure:**
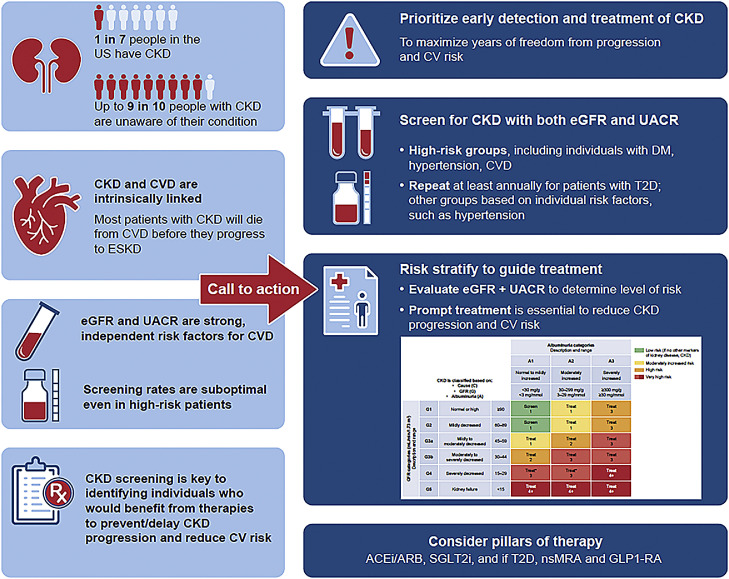
**Central Illustration. Call to Action for the Detection and Treatment of CKD.** Albuminuria and GFR grid reflect the risk of CKD progression by intensity of coloring (green, yellow, orange, red, and deep red). The numbers in the boxes are a guide to the frequency of monitoring (number of times per year). ACEi: angiotensin-converting enzyme inhibitor; ACR: albumin-to-creatinine ratio; ARB: angiotensin receptor blocker; CKD: chronic kidney disease; CVD: cardiovascular disease, DM: diabetes mellitus; eGFR: estimated glomerular filtration rate; eGFRcr: creatinine-based estimated glomerular filtration rate; ESKD: end-stage kidney disease; GLP-1 RA: glucagon-like peptide-1 receptor agonist; nsMRA: non-steroidal mineralocorticoid receptor antagonist; SGLT2i: sodium-glucose cotransporter 2 inhibitor; T2D: type 2 diabetes; UACR: urine albumin-to-creatinine ratio.

## 1 Introduction

Chronic kidney disease (CKD) is defined as the presence of abnormalities of kidney structure or function for a minimum of 3 months, as demonstrated by an estimated glomerular filtration rate (eGFR) of <60 mL/min/1.73 m^2^ and/or markers of kidney damage including albuminuria as assessed by a urine albumin-to-creatinine ratio (UACR) ≥30 mg/g [1,2].Evaluation of both eGFR and UACR levels is essential to determine the level of risk and to guide appropriate treatment [1,3].

CKD has a high global prevalence and affects around 1 in 7 adults in the United States [4]. It is of concern that 9 in 10 adults with CKD are unaware that they have the condition, even among individuals with advanced disease [4]. CKD is associated with increased morbidity and mortality [4,5]; therefore, early detection and treatment are crucial. Furthermore, CKD is a strong, independent risk factor for atherosclerotic cardiovascular disease (CVD) [[6], [7], [8]]. There is also a bidirectional relationship between CKD and heart failure (HF), with the presence of 1 condition increasing the development and progression of the other [9]. The coexistence of CVD and CKD has important prognostic implications, and many patients with CKD are more likely to die from CVD than progress to end-stage kidney disease (ESKD) [6,10]. Even in some conditions such as IgA nephropathy where ESKD commonly precedes CVD, CVD risk remains high [11]. Accordingly, reduction of CV risk must be a priority. This involves early detection and treatment of CKD to maximize years of freedom from disease progression and complications, including reducing CV risk. In addition to morbidity and mortality, CKD poses a significant global economic burden, with costs rising substantially in line with disease progression [5,12,13]. The economic costs of CKD comprise the cost of treatments, including dialysis and transplant, as well as costs arising from comorbidities, particularly CVD, and associated costs of care and rehabilitation, in addition to societal costs such as lost productivity.

## 2 CKD and cardiovascular risk are intrinsically linked

Both reduced eGFR levels and elevated UACR are strongly associated with an increased risk of cardiovascular (CV) events, CKD progression, and all-cause mortality [3,[14], [15], [16], [17]]. These associations persist irrespective of age or sex, or the presence of diabetes or CVD [16]. Abnormalities in eGFR and UACR levels act as independent risk factors; therefore, assessment of both is essential to evaluate CV risk [1,3]. Reduced eGFR has an independent, graded association with risk of all-cause mortality, CV events, and hospitalization [18]. The rate of decline in eGFR over time also has prognostic significance: a large negative eGFR slope (change at 1 year from baseline worse than −10 mL/min/1.73m^2^) has been linked with an approximately 2-fold higher risk of CVD, irrespective of baseline eGFR level [2].

It is important to note that eGFR declines with healthy aging and that the Kidney Disease Improving Global Outcomes (KDIGO) definition of CKD does not take this into account [19,20]. As a consequence, the prevalence of CKD in elderly populations may be over-estimated, while potentially being missed in younger individuals [19,20]. This has led to the suggestion that there should be an age-adapted definition of CKD based on different eGFR thresholds for defined age groups, with an eGFR of <75 mL/min/1.73 m^2^ being proposed as a more appropriate threshold for CKD diagnosis in individuals aged <40 years, < 60 mL/min/1.73 m^2^ for those aged 40–65 years and <45 mL/min/1.73m2 >65 years [20]. Further research is needed to define the role of age-adapted eGFR thresholds in the definition of CKD [19].

While the effect on declining eGFR on CVD risk is diminished by aging, albuminuria is a strong, independent risk factor for CVD that is independent of age [19]. However the importance of assessing albuminuria as a predictor of CVD risk and mortality is not always recognized in clinical practice [1,16,21,22]. A large population-based cohort study showed that elevated UACR levels were associated with the presence of subclinical atherosclerosis, and increased risks of CV events and all-cause mortality compared with individuals with normal levels [23]. Even high-normal levels of UACR (>10<30 mg/g) are associated with an increased likelihood of CV mortality and all-cause mortality [16,24]. Another study showed that high-normal UACR levels (9.5–29.78 mg/g) were associated with increased risk of atherosclerosis (indicated by severe aortic arterial calcification), with the upper tertile of UACR showing a 1.5 times higher risk than the lower tertile [25]. The importance of early diagnosis of CKD is underscored by its identification as a risk-enhancing factor for atherosclerotic CVD (ASCVD) by the American College of Cardiology/American Heart Association in their clinical guideline on the primary prevention of CVD [26]. The presence of CKD is also an important prognostic indicator in the context of secondary prevention. In patients with established ASCVD, elevated UACR is more strongly associated with long-term outcomes than eGFR levels [27,28].

HF is also frequently associated with CKD and is found in nearly a quarter of patients with ESKD [5]. Conversely, in patients with HF, eGFR levels below 60 mL/min/1.73m^2^ have been demonstrated in 51 % and 55 % of patients with a reduced or preserved ejection fraction (HFrEF or HFpEF), respectively [29]. Importantly, the presence of a reduced eGFR is a predictor of all-cause mortality in patients with HF, with mortality increasing in line with declining eGFR levels [29]. In addition, UACR levels can predict the risk of hospitalization for HF in individuals with CKD [30].

## 3 Screening is crucial - using both eGFR and UACR

**Fig. 1:**
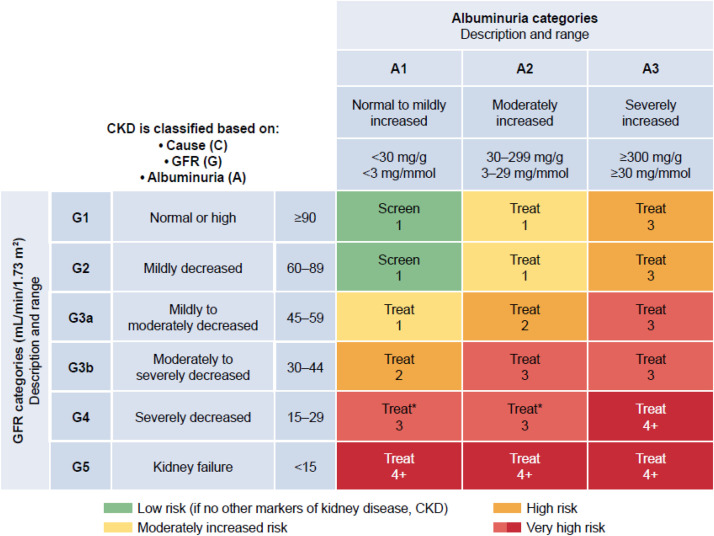
Frequency of monitoring glomerular filtration rate (GFR) and albuminuria in people with chronic kidney disease (CKD) [1]. Albuminuria and GFR grid reflect the risk of CKD progression by intensity of coloring (green, yellow, orange, red, and deep red). The numbers in the boxes are a guide to the frequency of monitoring (number of times per year). ACR: albumin-to-creatinine ratio; CKD: chronic kidney disease; eGFRcr: creatinine-based estimated glomerular filtration rate.

Early detection of CKD provides an important opportunity for intervention aimed at delaying progression and other adverse outcomes including CVD [3]. At present there is no consensus on the role of population screening or targeted screening programs for CKD [31,32]. However, in the setting of T2D, a consensus report from the American Diabetes Association (ADA) and Kidney Disease: Improving Global Outcomes (KDIGO) recommends that screening for CKD should start as soon as T2D is diagnosed and continued annually thereafter [33]. Furthermore, the 2024 KDIGO clinical practice guidelines recommend screening of high-risk groups, including individuals with diabetes, hypertension, and CVD [1,3]. KDIGO published a series of ‘heatmaps’, including a color-coded depiction of eGFR and UACR categories and the associated risk of CKD progression (Fig. 1), in addition to heatmaps showing the risk of other outcomes, including all-cause and CV mortality, myocardial infarction, stroke, and HF [1,3]. Evaluation of both eGFR and UACR is essential for effective risk stratification of patients and informs patient education, approaches to management, and the need for specialty referral. The timing of repeat eGFR and UACR testing should be based on patient characteristics such as the risk of CKD progression [1]. For patients with type 2 diabetes (T2D) and established CKD, the ADA advise that eGFR and UACR are monitored 1–4 times annually, depending on CKD stage [34]. As noted previously, it is possible that the use of age-adjusted eGFR values could avoid overestimating the risk of CKD progression and CVD in individuals of older age [17,19,20]. However, further research is needed before age-adjusted values are considered for incorporation into clinical practice [19].

## 4 Clinical note: screening for albuminuria

Dipstick urine assays are simple and inexpensive but less sensitive than UACR for detecting clinically important albuminuria [1,3]. Ideally, assessment of UACR should be performed with an early-morning urine sample, although a random spot urine sample is also acceptable if a morning sample is difficult to obtain [1,33,35]. While the clinical role of albuminuria point-of-care testing for identification and monitoring of CKD has yet to be determined, it is likely that these technologies will play a future role by addressing barriers around access to laboratory testing in the community [36].

As recent evidence indicates that CV risk increases below the standard cut-off value of 30 mg/g, a lower UACR cut-off (such as 10 mg/g) may provide a more sensitive method for identifying patients at elevated CV risk [16,37]. For the diagnosis of CKD, individuals should receive at least 2 UACR measurements at least 3 months apart [1]. Reduction of albuminuria by at least 30 % from baseline (or to <300 mg/g creatinine) has been shown to be associated with improvements in kidney and CV outcomes [38,39]. Based on this finding, the ADA advise that treatments aimed at reducing albuminuria should be titrated to maximize reductions in UACR levels [34]. Elevation of UACR, even within the normal range, is being increasingly recognized as a modifiable risk factor for CVD, particularly in patients with pre-existing coronary artery disease or T2D, underscoring the clinical importance of screening [[40], [41], [42]].

## 5 Intervene early with effective treatment

Involvement of a multidisciplinary team of healthcare professionals is essential for ensuring comprehensive care of people with CKD [1]. A patient-centered multidisciplinary care approach is recommended, which includes dietary counseling, medication management, education, and counseling about treatment choices, in addition to psychological and social care [1]. A range of therapies is now available to reduce the risk of CKD progression and CV events for people with CKD. Prompt treatment should be prioritized to minimize the development of irreversible pathological progression, and reduce the risk of subsequent morbidity and mortality [1,43]. Treatments include renin–angiotensin system inhibitors (RASi; angiotensin-converting enzyme inhibitors, ACEi; or angiotensin receptor blockers, ARB), sodium-glucose cotransporter 2 (SGLT2) inhibitors (SGLT2i), and for patients with T2D, non-steroidal mineralocorticoid receptor antagonists (nsMRA), and glucagon-like peptide-1 receptor agonists (GLP-1 RA) [44]. Current approaches to management of CKD involve the use of multiple evidence-based therapies that target different pathological pathways of action to reduce cardiorenal risk [44]. This ‘pillars of care’ approach to treatment can address multiple mechanisms of CKD progression as well as provide CV benefits [1,34,45].

**Fig. 2:**
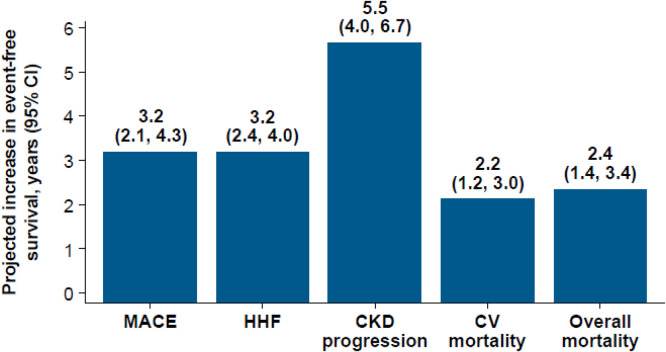
Treatment benefits of combination therapy with SGLT2i, GLP-1 RA, and nsMRA when added to RAS blockade in patients with T2D and albuminuria (UACR ≥30 mg/g) versus RAS blockade alone (conventional therapy) [46]. Projected increases in event-free survival (years, 95% CI) in a 50-year-old patient with T2D and albuminuria initiating combination therapy versus standard care. CKD progression is defined as doubling of serum creatinine, kidney failure, or death resulting from kidney failure. CI: confidence interval; CKD: chronic kidney disease; CV: cardiovascular; HHF: hospitalization for heart failure; MACE: major adverse cardiovascular event.

Striking reductions in cardiorenal risk may be achieved by combining appropriate evidence-based therapies, as exemplified by an actuarial analysis of data from large-scale clinical trials of SGLT2i, GLP-1 RAs, and nsMRA in people with T2D and albuminuria (UACR ≥30 mg/g) [46]. Compared with conventional treatment with RAS blockade and risk factor control, the addition of combination therapy with SGLT2i, GLP-1 RA, and nsMRA was associated with a 35 % reduction in major adverse cardiovascular events (MACE, nonfatal myocardial infarction, nonfatal stroke, or CV death) [1]. For a 50-year-old patient with T2D and albuminuria initiating combination therapy, MACE-free survival increased to 21.1 years versus 17.9 years for standard care (Fig. 2). Projected gains in event-free survival were also observed for hospitalization for HF (HHF), CKD progression, CV death, and all-cause mortality. Lifetime gains in event-free and overall survival were observed across the included age range (50–80 years) [46].

New developments in the treatment landscape of CKD provide major opportunities to delay or reduce the occurrence of CV events and premature death with combined use of SGLT2i, GLP-1RA, and nsMRA for patients with T2D at high cardiorenal risk [21,46,47]. A recent example of the incremental benefit of combining treatments has been shown in a clinical trial of the combination of SGLT2i and nsMRA in patients with CKD and T2D, where initial therapy with the combination led to greater reduction in UACR than either treatment alone [48]. New treatment combinations, including an aldosterone synthase inhibitor or an endothelin receptor antagonist, to be added to RASi and SGLT2i are being evaluated in ongoing trials [[49], [50], [51]] and have the potential to further expand the treatment options for patients with CKD [44].

## 6 Prioritize CKD diagnosis and treatment: a call to action

There is evidence to suggest underuse of dual screening for CKD involving both eGFR and UACR in primary care in the United States [[52], [53], [54]]. Screening rates are suboptimal even among high-risk patients, including individuals with diabetes and hypertension [[52], [53], [54]]. Although only around 50 % of patients with diabetes receive UACR assessment, the situation is worse for patients with hypertension (without diabetes), with only around 5 % of patients receiving screening for albuminuria [52,54]. Furthermore, low rates of UACR testing have been observed even among patients with an existing diagnosis of CKD based on eGFR [55]. These findings indicate substantial room for improvement in identification of abnormalities in eGFR and UACR levels, which can be considered as modifiable risk factors for CKD progression and adverse CV outcomes. We raise a call to action to address these unmet needs and improve the standard of care for early diagnosis and management of CKD, to minimize the risk of disease progression and complications, reduce CV risk and ultimately improve patient outcomes (**Central illustration**).

CKD should receive greater attention in health policy decision-making, and national strategies for addressing CKD are central to achieve changes aimed at improving kidney health and, in turn, likely to reduce the risk of CVD (Table) [56,57]. Strategies aimed at improving screening and early identification of CKD and adherence to evidence-based treatments require policy measures to support the development of initiatives by ensuring financial support and equitable access to healthcare [58]. Measures include facilitating continuity of care through a holistic approach involving liaison between patients, primary care and specialist clinicians, and improving access to healthcare. The development of environmentally sustainable approaches to the management of CKD should also be a key consideration in the development of health policy and practice [58,59]. The International Society of Nephrology and the International Federation of Kidney Foundations – World Kidney Alliance have jointly initiated a global health awareness campaign, which takes place as an annual World Kidney Day advocating action on prevention, awareness, treatment access, and environmental risk reduction [59]. The aim of this initiative is to encourage governments, health systems, industry, and communities to prioritize prevention, early detection, and timely management of CKD, and promote equitable access to treatments. Furthermore, in May 2025, the World Health Organization made a historic decision to adopt a resolution to promote kidney health and prioritize the prevention and control of CKD with the aim of reducing the global burden of the condition [58,60]

**Table 1:** Implementation strategies for early diagnosis and treatment of CKD.

Unmet need	Implementation Strategies
Raise awareness of need for CKD screening and treatment	• Collaboration of professional societies for kidney, heart, diabetes, hypertension ∘ World Kidney Day Campaign [59] • Educational initiatives for patients and families
Increase early diagnosis of CKD	• Facilitate early diagnosis, classification and guideline-directed management ∘ Electronic medical record alerts ∘ Clinician tools e.g. National Kidney Foundation [62] • Ongoing training of clinicians to keep up to date with clinical guidelines
Optimize treatment outcomes	• Patient monitoring in line with individual level of risk • Individualized approach to treatment based on patient characteristics • Active patient engagement in treatment plans • Clinical decision support tools ∘ Kidney Failure Risk Equation • Collaboration between clinical specialties to provide integrated care ∘ Nephrologists, endocrinologists, cardiologists, primary care • Specialist clinics ∘ Cardiorenal/ CKM clinics
Ensure equity of access to treatments	• Health policy decision-making and national strategies ∘ Guidance based on clinical practice guidelines, e.g. KDIGO [1], ADA [34], ACC/AHA [26] ∘ Ensure access to treatments in an affordable manor, e.g. WHO initiative [60]

ADA, American Diabetes Association; ACC/AHA, American College of Cardiology/ American Hypertension Association; CKM, cardio-kidney-metabolic, KDIGO, Kidney Disease: Improving Global Outcomes; WHO, World Health Organization.

The success of a CKD screening program will require commitment from local healthcare professionals, including primary care physicians (PCPs), cardiologists, endocrinologists, nephrologists, and geriatricians [1,3]. PCPs play a key role in the screening and treatment of early-stage CKD, in partnership with frontline health workers and allied healthcare professionals [1,3]. Cardiologists also should lead the way for preventive efforts. Since patients with ASCVD and/or HF have a high prevalence of CKD, and since CVD is the leading cause of death among patients with CKD, cardiologists should play a pivotal role in screening for CKD and implementing these pillars of therapy that reduce the risk of both CKD progression and CVD events. Furthermore, cardiologists will have the opportunity to implement preventive measures for their patients with hypertension or other CV risk factors. Clinicians need to stay up to date with practice guidelines; this can be facilitated by ongoing training. Decision support tools such as electronic medical record alerts can help to identify suitable individuals for screening [61]. The National Kidney Foundation provides tools for PCPs and cardiologists to assist with appropriate screening, identification, classification, and management of CKD [62]. In tandem, patients need to be encouraged to become active participants in their care by ensuring they receive education about their condition, treatment options, and approaches to self-care management [1,3].

## 7 Take-home points

• Increased recognition of CKD as a modifiable CV risk factor is needed to address the burden of CVD in the population.
• Since most patients with CKD will die from CVD before they progress to ESKD, reduction of CV risk must be a priority. This involves early detection and treatment of CKD to maximize years of freedom from disease progression and complications, including reducing CV risk.
• While there is no current consensus on the recommended approach to screening, targeting high-risk groups, including individuals with diabetes, hypertension, and CVD, is recommended by the latest clinical practice guidelines from KDIGO. For patients with T2D, joint guidelines from the ADA and KDIGO recommend screening for CKD at diagnosis and continued annually thereafter.
• Assessment of both eGFR and UACR levels is necessary to determine the level of risk and to guide appropriate treatment.
• Opportunities to diagnose and treat CKD early must be maximized across clinical settings including cardiology, rather than being restricted to primary care or nephrology.
• Effective therapies are available that can prevent or delay CKD onset and progression, as well as reduce CV risk. It is imperative that patients receive appropriate treatment as early as possible.
• A holistic, multidisciplinary approach to the care of people with CKD is essential to ensure continuity of care. This may involve a range of clinicians, including cardiologists, nephrologists, endocrinologists, and PCPs.
• The time is now to focus on early diagnosis and management of CKD with the aim of preventing disease progression and complications, reducing CV risk, and improving patient outcomes.

## Glossary

ACEi, angiotensin-converting enzyme inhibitor; ACR, albumin-to-creatinine ratio; ADA, American Diabetes Association; ARB, angiotensin receptor blocker; ASCVD, atherosclerotic cardiovascular disease; CI, confidence interval; CKD, chronic kidney disease; CV, cardiovascular; CVD, cardiovascular disease; DM, diabetes mellitus; eGFR, estimated glomerular filtration rate; eGFRcr: creatinine-based estimated glomerular filtration rate; GLP-1RA, glucagon-like peptide-1 receptor agonist; HF, heart failure; HFpEF, heart failure with a preserved ejection fraction; HFrEF, heart failure with a reduced ejection fraction; HHF, hospitalization for heart failure; KDIGO, Kidney Disease: Improving Global Outcomes; MACE, major adverse cardiovascular event; nsMRA, nonsteroidal mineralocorticoid receptor antagonist; PCP, primary care physician; RASi, renin–angiotensin system inhibitor; SGLT2, sodium-glucose cotransporter 2; SGLT2i, sodium-glucose cotransporter 2 inhibitor; T2D, type 2 diabetes; UACR, urinary albumin-to-creatinine ratio.

## Funding

This work was supported by Boehringer Ingelheim (BI). BI was given the opportunity to review the manuscript for medical and scientific accuracy as well as intellectual property considerations.

## CRediT authorship contribution statement

All authors were involved inthe conceptualization of the article and were involved in all stages of drafting and critical review. All authors provided review and approval of the final draft.

## Data statement

Not applicable

## Ethical review statement

Not applicable

## Author agreement

**Chronic kidney disease screening to reduce cardiovascular risk: a call to action**

Erin D. Michos, David Cherney, Pam Kushner

All authors have seen and approved the final version of the manuscript being submitted. The article is the authors' original work, has not received prior publication, and is not under consideration for publication elsewhere.

## CRediT authorship contribution statement

**Erin D. Michos:** Writing – review & editing, Supervision. **David Cherney:** Writing – review & editing. **Pam Kushner:** Writing – review & editing.

## Declaration of competing interest

Erin Michos has served as a consultant for Arrowhead, Bayer, Boehringer Ingelheim, Edwards Lifesciences, Eli Lilly, Ionis, Merck, New Amsterdam, Novartis, Novo Nordisk, and Pfizer.

David Cherney has received honoraria from AbbVie, Altimmune, AMGEN, AstraZeneca, Bayer, Biobridge, BMS, Boehringer Ingelheim-Lilly, CSL-Behring, Gilead, GSK, Inversago, Janssen, Lexicon, Maze, Merck, Mitsubishi-Tanabe, Novartis, Novo Nordisk, Otsuka, Prometic, Roche, Sanofi, Vantage, and Youngene, and has received operational funding for clinical trials from AstraZeneca, Bayer, Boehringer Ingelheim-Lilly, CSL-Behring, Janssen, Lexicon, Merck, Novo Nordisk, and Sanofi.

Pam Kushner has served as a consultant and speaker for AstraZeneca, Bayer, Boehringer Ingelheim, Eli Lilly, and Janssen. She has served as a consultant for Amgen, Novo Nordisk, and Verdiva.

## References

[1] (2024). Kidney Disease: improving global outcomes (KDIGO) CKD Work Group. KDIGO 2024 clinical practice guideline for the evaluation and management of chronic kidney disease. Kidney Int.

[2] Fujisawa T., Suzuki S., Arita T. (2021). Decline in eGFR over time and incidence of cardiovascular events: Shinken Database analysis. J Cardiol.

[3] Shlipak M.G., Tummalapalli S.L., Boulware L.E. (2021). The case for early identification and intervention of chronic kidney disease: conclusions from a Kidney Disease: Improving Global Outcomes (KDIGO) controversies conference. Kidney Int.

[4] US Centers for Disease Control and Prevention (2025). https://nccd.cdc.gov/CKD/default.aspx.

[5] United States Renal Data System (2024). https://usrds-adr.niddk.nih.gov/2024.

[6] Jankowski J., Floege J., Fliser D., Bohm M., Marx N. (2021). Cardiovascular disease in chronic kidney disease: pathophysiological insights and therapeutic options. Circulation.

[7] Coyle M., Flaherty G., Jennings C. (2021). A critical review of chronic kidney disease as a risk factor for coronary artery disease. Int J Cardiol Heart Vasc.

[8] Sarnak M.J., Levey A.S., Schoolwerth A.C. (2003). Kidney disease as a risk factor for development of cardiovascular disease: a statement from the American Heart Association Councils on Kidney in Cardiovascular Disease, High Blood Pressure Research. Clin Cardiol Epidemiol Prev Hypertens.

[9] House A.A., Wanner C., Sarnak M.J. (2019). Heart failure in chronic kidney disease: conclusions from a kidney disease: improving global outcomes (KDIGO) controversies conference. Kidney Int.

[10] Foley R.N., Murray A.M., Li S. (2005). Chronic kidney disease and the risk for cardiovascular disease, renal replacement, and death in the United States Medicare population, 1998 to 1999. J Am Soc Nephrol.

[11] Glassock R.J. (2019). Mortality risk in IgA nephropathy. J Am Soc Nephrol.

[12] Jha V., Al-Ghamdi SMG, Li G. (2023). Global economic burden associated with chronic kidney disease: a pragmatic review of medical costs for the inside CKD research programme. Adv Ther.

[13] Elshahat S., Cockwell P., Maxwell A.P., Griffin M., O'Brien T., O'Neill C. (2020). The impact of chronic kidney disease on developed countries from a health economics perspective: a systematic scoping review. PLoS One.

[14] Thompson S., James M., Wiebe N. (2015). Cause of death in patients with reduced kidney function. J Am Soc Nephrol.

[15] Ohkuma T., Jun M., Chalmers J. (2019). Combination of changes in estimated GFR and albuminuria and the risk of major clinical outcomes. Clin J Am Soc Nephrol.

[16] Grams M.E., Coresh J., Writing Group for the CKD Prognosis Consortium (2023). Estimated glomerular filtration rate, albuminuria, and adverse outcomes: an individual-participant data meta-analysis. JAMA.

[17] Azegami T., Kaneko H., Okada A. (2024). Significance of eGFR and proteinuria for cardiovascular disease in individuals beyond 85 years of age. Nephrol Dial Transpl.

[18] Go A.S., Chertow G.M., Fan D., McCulloch C.E., Hsu C.Y. (2004). Chronic kidney disease and the risks of death, cardiovascular events, and hospitalization. N Engl J Med.

[19] Astley M.E., Chesnaye N.C., Gambaro G. (2025). Prevalence of reduced eGFR in European adults using KDIGO and age-adapted eGFR thresholds. Nephrol Dial Transpl.

[20] Delanaye P., Jager K.J., Bokenkamp A. (2019). CKD: a call for an age-adapted definition. J Am Soc Nephrol.

[21] Kotwal S.S., Perkovic V. (2024). Kidney disease as a cardiovascular disease priority. Circulation.

[22] De Nicola L., Correa-Rotter R., Navarro-Gonzalez J.F. (2024). Projecting the population level burden of CKD progression according to urine albumin-to-creatinine ratio categories. Kidney Int Rep.

[23] Liu S., Niu J., Wu S. (2021). Urinary albumin-to-creatinine ratio levels are associated with subclinical atherosclerosis and predict CVD events and all-cause deaths: a prospective analysis. BMJ Open.

[24] Inoue K., Streja E., Tsujimoto T., Kobayashi H. (2021). Urinary albumin-to-creatinine ratio within normal range and all-cause or cardiovascular mortality among U.S. adults enrolled in the NHANES during 1999-2015. Ann Epidemiol.

[25] Xue X., Li C., Chen D. (2024). A cross-sectional study investigating the relationship between urinary albumin creatinine ratio and abdominal aortic calcification in adults. Front Cardiovasc Med.

[26] Arnett D.K., Blumenthal R.S., Albert M.A. (2019). 2019 ACC/AHA guideline on the primary prevention of cardiovascular disease: a report of the American College of Cardiology/American Heart Association task force on clinical practice guidelines. Circulation.

[27] Zhu H., Yang C., Liu X. (2025). Urinary albumin-to-creatinine ratio as an independent predictor of long-term mortality in atherosclerotic cardiovascular disease patients: a propensity score-matched study: UACR and long-term mortality in ASCVD. Am J Prev Cardiol.

[28] Mok Y., Ballew S.H., Stacey R.B. (2021). Albuminuria and prognosis among individuals with atherosclerotic cardiovascular disease: the ARIC study. J Am Coll Cardiol.

[29] McAlister F.A., Ezekowitz J., Tarantini L. (2012). Renal dysfunction in patients with heart failure with preserved versus reduced ejection fraction: impact of the new Chronic Kidney Disease-Epidemiology Collaboration Group formula. Circ Heart Fail.

[30] Mehta R., Ning H., Bansal N. (2022). Ten-year risk-prediction equations for incident heart failure hospitalizations in chronic kidney disease: findings from the Chronic Renal Insufficiency Cohort study and the Multi-ethnic Study of Atherosclerosis. J Card Fail.

[31] Cordero L., Ortiz A. (2025). Albuminuria-based universal screening for CKD should be implemented now in high-income countries. Kidney Int.

[32] Wong G., van Zwieten A. (2025). To screen or not to screen: why universal CKD testing is not a goer!. Kidney Int.

[33] de Boer I.H., Khunti K., Sadusky T. (2022). Diabetes management in chronic kidney disease: a consensus report by the American Diabetes Association (ADA) and Kidney Disease: Improving Global Outcomes (KDIGO). Diabetes Care.

[34] American Diabetes Association Professional Practice Committee (2025). 11. Chronic kidney Disease and Risk Management: Standards of Care in Diabetes-2025. Diabetes Care.

[35] Alicic R., Nicholas S.B. (2022). Diabetic kidney disease back in focus: management field guide for health care professionals in the 21st century. Mayo Clin Proc.

[36] Nasuuna E.M., Kalyesubula R., Tomlinson L.A. (2024). Diagnostic performance of an albuminuria point-of-care test in screening for chronic kidney disease among young people living with HIV in Uganda: a cross-sectional study. BMJ Open.

[37] Yang Z.W., Fu Y.B., Wei X.B. (2023). Optimal threshold of urinary albumin-to-creatinine ratio (UACR) for predicting long-term cardiovascular and noncardiovascular mortality. Int Urol Nephrol.

[38] Coresh J., Heerspink H.J.L., Sang Y. (2019). Change in albuminuria and subsequent risk of end-stage kidney disease: an individual participant-level consortium meta-analysis of observational studies. Lancet Diabetes Endocrinol.

[39] Tangri N., Singh R., Chen Y. (2025). Change in urine albumin-to-creatinine ratio and clinical outcomes in patients with chronic kidney disease and type 2 diabetes. BMJ Open Diabetes Res Care.

[40] Lin X., Song W., Zhou Y. (2023). Elevated urine albumin creatinine ratio increases cardiovascular mortality in coronary artery disease patients with or without type 2 diabetes mellitus: a multicenter retrospective study. Cardiovasc Diabetol.

[41] Zannad F., McGuire D.K., Ortiz A. (2025). Treatment strategies to reduce cardiovascular risk in persons with chronic kidney disease and type 2 diabetes. J Intern Med.

[42] Mahemuti N., Zou J., Liu C., Xiao Z., Liang F., Yang X. (2023). Urinary albumin-to-creatinine ratio in normal range, cardiovascular health, and all-cause mortality. JAMA Netw Open.

[43] Handelsman Y., Butler J., Bakris G.L. (2023). Early intervention and intensive management of patients with diabetes, cardiorenal, and metabolic diseases. J Diabetes Complicat.

[44] Neuen B.L., Yeung E.K., Rangaswami J., Vaduganathan M. (2025). Combination therapy as a new standard of care in diabetic and non-diabetic chronic kidney disease. Nephrol Dial Transpl.

[45] Handelsman Y., Anderson J.E., Bakris G.L. (2024). DCRM 2.0: multispecialty practice recommendations for the management of diabetes, cardiorenal, and metabolic diseases. Metabolism.

[46] Neuen B.L., Heerspink H.J.L., Vart P. (2024). Estimated lifetime cardiovascular, kidney, and mortality benefits of combination treatment with SGLT2 inhibitors, GLP-1 receptor agonists, and nonsteroidal MRA compared with conventional care in patients with type 2 diabetes and albuminuria. Circulation.

[47] Bozkurt B., Rossignol P., Vassalotti J.A. (2025). Albuminuria as a diagnostic criterion and a therapeutic target in heart failure and other cardiovascular disease. Eur J Heart Fail Epub ahead print.

[48] Agarwal R., Green J.B., Heerspink H.J.L. (2025). Finerenone with empagliflozin in chronic kidney disease and type 2 diabetes. N Engl J Med.

[49] AstraZeneca (Accessed 17 July 2025). A phase III study to investigate the efficacy and safety of baxdrostat in combination with dapagliflozin on CKD progression in participants with CKD and high blood pressure. https://clinicaltrials.gov/study/NCT06268873.

[50] Judge P.K., Tuttle K.R., Staplin N. (2025). The potential for improving cardio-renal outcomes in chronic kidney disease with the aldosterone synthase inhibitor vicadrostat (BI 690517): a rationale for the EASi-KIDNEY trial. Nephrol Dial Transpl.

[51] AstraZeneca (Accessed 16 July 2025). Study to investigate efficacy, safety, and tolerability of zibotentan/dapagliflozin compared to dapagliflozin in participants with chronic kidney disease and high proteinuria (ZENITH high proteinuria). https://clinicaltrials.gov/study/NCT06087835.

[52] Stempniewicz N., Vassalotti J.A., Cuddeback J.K. (2021). Chronic kidney disease testing among primary care patients with type 2 diabetes across 24 US. health care organizations. Diabetes Care.

[53] Alfego D., Ennis J., Gillespie B. (2021). Chronic kidney disease testing among at-risk adults in the U.S. remains low: real-world evidence from a national laboratory database. Diabetes Care.

[54] Chu C.D., Xia F., Du Y. (2023). Estimated prevalence and testing for albuminuria in US adults at risk for chronic kidney disease. JAMA Netw Open.

[55] Tangri N., Peach E.J., Franzen S., Barone S., Kushner P.R (2023). Patient management and clinical outcomes associated with a recorded diagnosis of stage 3 chronic kidney disease: the REVEAL-CKD study. Adv Ther.

[56] Chronic Kidney Disease Collaboration G.B.D. (2020). Global, regional, and national burden of chronic kidney disease, 1990-2017: a systematic analysis for the Global Burden of Disease Study 2017. Lancet.

[57] Neuen B.L., Bello A.K., Levin A. (2023). National health policies and strategies for addressing chronic kidney disease: data from the International Society of Nephrology Global Kidney Health Atlas. PLOS Glob Public Health.

[58] Faucon A.L. (2025). CKD beyond the clinic: a holistic projection of its global burden. Kidney Int Rep.

[59] (2025). The International Society of Nephrology and the International Federation of Kidney Foundations – World Kidney Alliance. World Kidney Day Campaign.

[60] World Health Organization. Reducing the burden of noncommunicable diseases through promotion of kidney health and strengthening prevention and control of kidney disease. https://apps.who.int/gb/ebwha/pdf_files/EB156/B156_(20)-en.pdf; Accessed 20 November 2025.

[61] Alexiuk M., Elgubtan H., Tangri N. (2024). Clinical decision support tools in the electronic medical record. Kidney Int Rep.

[62] Clinician Tools National Kidney Foundation. https://www.kidney.org/professionals/tools. Accessed 17 July 2025.

